# Anthropometric measures in relation to Basal Cell Carcinoma: a longitudinal study

**DOI:** 10.1186/1471-2407-6-82

**Published:** 2006-03-27

**Authors:** Catherine M Olsen, Maria Celia Hughes, Nirmala Pandeya, Adèle C Green

**Affiliations:** 1 Cancer and Population studies group, Queensland Institute of Medical Research, Brisbane, Australia; 2 School of Population Health, University of Queensland, Brisbane, Australia

## Abstract

**Background:**

The relationship between anthropometric indices and risk of basal cell carcinoma (BCC) is largely unknown. We aimed to examine the association between anthropometric measures and development of BCC and to demonstrate whether adherence to World Health Organisation guidelines for body mass index, waist circumference, and waist/hip ratio was associated with risk of BCC, independent of sun exposure.

**Methods:**

Study participants were participants in a community-based skin cancer prevention trial in Nambour, a town in southeast Queensland (latitude 26°S). In 1992, height, weight, and waist and hip circumferences were measured for all 1621 participants and weight was remeasured at the end of the trial in 1996. Prevalence proportion ratios were calculated using a log-binomial model to estimate the risk of BCC prior to or prevalent in 1992, while Poisson regression with robust error variances was used to estimate the relative risk of BCC during the follow-up period.

**Results:**

At baseline, 94 participants had a current BCC, and 202 had a history of BCC. During the 5-year follow-up period, 179 participants developed one or more new BCCs. We found no significant association between any of the anthropometric measures or indices and risk of BCC after controlling for potential confounding factors including sun exposure. There was a suggestion that short-term weight gain may increase the risk of developing BCC for women only.

**Conclusion:**

Adherence to World Health Organisation guidelines for body mass index, waist circumference and waist/hip ratio is not significantly associated with occurrence of basal cell carcinomas of the skin.

## Background

To date only two previous studies have investigated the association between anthropometric measures and basal cell carcinoma (BCC) [1,2]. Sahl *et al *(1995) in a small case-control study based on 46 cases reported a positive association between weight and BCC, whilst Milan *et al *(2003) in a nested case-control study of disease-discordant same-sex twin pairs based on 333 cases of BCC, found no relationship between BMI and risk of BCC. In neither study did the authors consider potential confounders such as phenotypic characteristics and sun exposure.

Anthropometric measures such as height, weight and body mass index (BMI) are associated with the development of certain malignancies [3]. Increasing height has been associated with increased risk of cancer of the breast, colon and prostate [3-5]. Obesity has been consistently associated with an increased risk of cancers of the colon, breast (in postmenopausal women), endometrium, kidney, oesophagus, and gastric cardia [6].

A number of hypotheses have been put forward to explain the associations of anthropometric measures and cancer. The first is that increasing height and/or weight correlate directly with the total number of cells that can undergo malignant transformation [7]. The second is that both adult height and cancer incidence are directly related to caloric intake in early life, implicating positive energy balance. Animal studies have found that reduced caloric intake during development reduces the future risk of malignancy [8,9] and one study in humans also supported this hypothesis [10]. The association between height, weight and cancer may be mediated via alterations in the metabolism of endogenous hormones [11,12]. Obesity may also result in long-term storage of toxins, medications and vitamins in adipose tissue [13] or modify immune function [14].

We examined the association between anthropometric measures and indices and risk of BCC in a representative sample of people aged 25 to 75 years living in the township of Nambour, Queensland, who were followed up prospectively for a period of 4.5 years from early 1992 to late 1996. Specifically we aimed to demonstrate whether or not adherence to World Health Organisation guidelines for BMI, waist circumference and waist/hip ratio was associated with development of BCC, independent of sun exposure.

## Methods

Participants were 1621 residents of Nambour, a subtropical town in South-East Queensland (latitude 26°S), who were originally randomly selected from the electoral roll (enrolment is compulsory) and who were participating in the Nambour Skin Cancer Prevention Trial between 1992 and 1996 to evaluate the preventive effects of sunscreen use and/or beta-carotene supplementation. Detailed descriptions of the trial and its outcomes are reported elsewhere [15,16]. Participants gave written informed consent at the beginning of the trial. At baseline in 1992, height, weight, waist and hip circumference were measured. Weight was re-measured at the end of the field trial (1996). In addition, personal information was collected at interview including skin colour, eye colour, hair colour, propensity of the skin to sunburn and number of sunburns, as well as outdoor exposure during occupation and leisure (classified as mainly indoors or outdoors, or a mixture of indoors and outdoors). All anthropometric measurements were obtained by trained nurses or nutritionists using standard procedures.

Dermatologists performed full skin examinations of participants in 1992 and again in 1994 and 1996 for the majority of participants, including some who had otherwise withdrawn. In 1996, 124 people who had missed the 1994 examination were again examined by a dermatologist. Every participant had at least one follow-up examination with a dermatologist in either 1994 or 1996. All lesions clinically diagnosed as BCC were biopsied for histologic confirmation by a single dermatopathologist. In addition, participants were followed-up every 3 months and any new cancers treated by the participant's General Practitioner were documented and later validated against histological records. Finally and with participants' consent, independent pathology laboratories throughout Queensland provided reports on all skin cancers diagnosed during the entire trial period for cross-checking. History of BCC or squamous cell carcinoma (SCC) prior to 1986 was obtained from a prevalence survey of skin cancer and actinic skin damage conducted in December 1986 [17], and BCCs and SCCs treated between 1986 and 1992 were monitored via surveys conducted between 1986 and 1992 [18,19].

The study was approved by the Queensland Institute of Medical Research ethics committee and abided by the Declaration of Helsinki on research on human subjects.

### Data analysis

Weight and height were expressed as quartiles of the distribution for the entire at-risk cohort, while BMI, waist circumference and waist/hip ratios were classified using World Health Organisation (WHO) definitions of obesity. The WHO BMI classification for adults was based on results of studies that linked morbidity and mortality to body weight [20] (<18.5 "underweight"; 18.5–24.9 "normal weight"; 25–29.9 "overweight"; and ≥ 30 obese). Waist circumference was categorised using the sex-specific WHO cut-offs for risk of metabolic complications in Caucasians [21]. Waist circumference measurements of < 94 cm for males and <80 cm for females were classified as 'desirable'; 94–102 cm for males and 80–88 cm for females were classified as 'increased obesity-associated risk'; and >102 cm for males and >88 cm for females were classified as 'substantially increased obesity-associated risk'. Waist/hip ratio was divided into two sex-specific categories, since high ratios (>1.0 in men and > 0.85 in women) is taken to indicate abdominal fat accumulation [22].

Two outcomes were examined: presence of one or more BCCs prior to the baseline survey or prevalent at the baseline survey in 1992 ('prevalent' BCC); and incidence of one or more histologically confirmed BCCs between the baseline survey in 1992 and 1996 ('incident' BCC).

Prevalence proportion ratios (PPR) with 95% confidence intervals (95% CI) were calculated as estimates of the risk of prevalent BCC associated with weight quartiles, height quartiles, BMI category, waist circumference and waist/hip ratio category, using the lowest category of each as the reference category. A multiplicative generalized linear model with logarithmic link function and binomial distribution function [23] was used to adjust for age and potential confounders such as education, smoking status, skin colour, hair colour, skin type, occupation type, leisure type, number of painful sunburns in lifetime, and use of hormone replacement therapy. Only those factors that changed the point estimate by >10% were included in the final models. Occupational exposure was taken to indicate cumulative sun exposure and leisure-time exposure, as indicating an irregular sun exposure pattern. Height was assessed as a potential confounder in the analyses for weight, waist circumference and waist/hip ratio categories, but it did not change the point estimate by >10% and so was not included in the final models.

To assess anthropometric measures at baseline in relation to incidence of BCC 1992–1996, relative risks (RR) for the binary outcome (new BCC 1992–1996 or no BCC ever) associated with weight quartiles, height quartiles, BMI, waist circumference and waist/hip ratio categories at baseline were calculated using a Poisson regression with robust error variance [24]. Multivariate Poisson regression was used to adjust for age and history of BCC, and other potential confounders as described for the analyses of prevalent BCC. Effect modification for both outcomes (prevalent and incident BCC) was assessed by comparing stratum-specific point estimates. If the stratum-specific point estimates appeared to differ, multiplicative terms were entered in to the model. A p-value of <0.05 for the multiplicative term was interpreted as significant effect modification. We also tested for homogeneity of the stratum-specific point estimate using chi-square test for homogeneity [25].

Possible relationship between change in weight 1992–1996 and incidence of BCC 1992–1996 was also investigated. Four arbitrary groups were created to describe weight change over the period 1992–1996: ≤ -4 kg, -4 kg to <4 kg, 4 kg to 10 kg, and >10 kg. The reference category for weight change was -4 kg to 4 kg. Relative risks (RR) with 95% confidence intervals were calculated using multivariate Poisson regression, adjusting for age, history of BCC, weight at baseline, hair colour and eye colour. To take into account the participants who had withdrawn without complete skin examination by a dermatologist in the follow-up period, total person-years were calculated for each participant, and the log-transformation of this variable was used as an off-set variable in the model.

Analyses were conducted using SAS (SAS Institute, Cary, North Carolina, USA).

## Results

Of the 1621 people (710 male and 911 female) who enrolled in the field trial at baseline, 238 participants (15%) withdrew without complete skin examination by a dermatologist in the follow-up period. Those with withdrew without follow-up examination were younger on average than the remaining participants (mean 47.0 vs 49.1 years), but were similar in all other respects [16]. In 1992 weight was recorded for 1233 participants, and height for 1112; both weight and height measurements were available for 1109 participants. Waist circumference and hip circumference was measured for 1221 participants, waist-hip ratio able to be calculated for 1220 participants. Weight in 1996 was recorded for 1271 participants; weight change 1992–1996 was available for 1079 participants.

Of this population sample, 55% had fair skin, 38% medium skin and only 7% olive or brown skin (p < 0.001). Most (68%) participants had a skin type that burned then tanned after acute sun exposure [26]. A high proportion of the sample (44%) reported that their main occupation was indoors, but in terms of leisure activities, only 15% of the sample reported that their main leisure activity was indoors.

Mean age at baseline was 50.7 years for men and 49.6 for women. At baseline 94 participants (6%) had a current BCC; 212 (13%) had been diagnosed with BCC in the past; and 82 participants (5%) had both a prevalent and a past history of BCC. During the follow-up period, 179 participants (12%) developed one or more new BCCs (97 (54%) of whom had no previous history of BCC). Of the women study participants, 1% were underweight (BMI<18.5), 32% were overweight (BMI 25–29.9), and 17% were obese (BMI ≥ 30). Few males (0.4%) were underweight, but 48% were overweight and a further 16% were obese.

Adjusted PPRs of prevalent BCC associated with anthropometric measures are shown in Table 1. For BMI the reference category was <25 (underweight and normal categories combined) as there were only 8 individuals in the underweight category. There was no association between prevalence of BCC and height for women or men; the PPR estimates for height were elevated only for the tallest people, but not significantly. Also no significant associations were seen between prevalence of BCC and weight, waist/hip ratio or waist circumference.

Similarly, anthropometric measures were unrelated to development of new BCCs. Women in the highest quartile of weight had a small increased risk (RR 1.4, 95% CI 0.9–2.4), but this was not statistically significant (Table 2). A separate analysis of incident BCC was performed for participants without a history of BCC, however the results did not differ significantly from those presented.

With respect to short-term weight change and development of BCC 1992–1996, weight gain showed a suggestive positive association with BCC for women (Table 2), but the point estimates were not statistically significant. Compared with women who gained or lost up to 4 kg, the risk of developing a BCC during the follow-up period was higher in those who gained 4–10 kg (RR 1.4, 95% CI 0.8–2.5), and in those who gained 10 kg or more (RR 1.7, 95% CI 0.5–5.6). For men, the RRs were only slightly elevated for those who gained 4–10 kg, and again this association was not statistically significant.

## Discussion

The relationship between anthropometric indices and occurrence of BCC has been little studied. To our knowledge, only two studies have investigated BCC in relation to anthropometric measures or indices [1,2]. The study by Milan *et al*. (2003) did not measure important potential confounders including phenotypic characteristics or sun exposure history, and although information on these potential confounders was collected in the study by Sahl *et al*. (1995), they did not consider them in their analyses which was limited to comparing the mean body weight of a small number of cases and controls (49 and 46 respectively). Although the current study was also not designed specifically to examine the association between anthropometric measures and BCC, information on a large number of other risk factors, including phenotypic characteristics and sun exposure history, was collected and these risk factors were assessed as possible confounders in the relationship between BCC and anthropometric measures.

In the present large prospective study, there were no significant associations between any of the anthropometric measures or indices and BCC, though a suggestive positive association between short term weight gain and development of BCC in women was observed. This implies that a stable body weight may be more favourable with respect to the development of BCC then short-term changes in body weight in this study population. It is unclear why weight gain might be associated with the development of BCC. Weight gain is a consequence of higher energy intake than energy expenditure over time, and availability or restriction of energy can modulate the cellular replication process. There is evidence from studies of different animal models that suggests caloric restriction inhibits cell proliferation [27] and carcinogenesis [28,29]. One study in human subjects also supported this hypothesis [10]. These data have been further supported by recent epidemiological studies that demonstrate the importance of energy balance and obesity in human cancer [3,5,30-32].

The association between weight gain and BCC, if confirmed by other studies, may be mediated via alterations in the metabolism of endogenous hormones (sex steroids, insulin and insulin-like growth factors) (that lead to a change in the normal balance between cell proliferation, differentiation and apoptosis [11,12,33]), or through reduction in immunity [14], although this hypothesis is based on results of animal studies.

For weight and BMI at baseline, no association was observed. Some research has suggested that adult weight gain may be a better variable to assess adiposity and its metabolic consequences than body weight itself [33], since weight gain largely reflects an increase in body fat, whereas weight reflects both lean and fat body mass. Height seemed to be associated inconsistently with an increased risk of developing BCC, but the point estimates were not significant, and there were no significant tests for trend. Tallness has been associated with increased risk of a number of other cancers, including cancers of the breast, colon, and prostate [3-5] and the same biological mechanisms as described above for weight gain have been proposed to explain these associations. It may also be possible that there is some behavioural correlate of body height related to sun exposure, such as participation in outdoor sporting activities [34], that increases the likelihood of developing BCC.

Strengths of this study include its large population sample with a high participation rate as well as its prospective design, and the ability to assess a large number of other risk factors as possible confounders of the association between anthropometric measures and BCC. Standardised methods for measuring weight and height, and comprehensive data on BCC incidence from 1992 -1996 were also features of this investigation. Our large prospective study did not have the limitations of the previous two studies. The case-control study by Sahl *et al*. (1995) was based on a very small number of clinic-based cases of BCC (less than 50), and thus it was limited in power, susceptible to selection bias due to non-representative cases and controls and to faulty recall of past weight and height measurements. Indeed both previous studies relied upon self-reported weight and height measurements. In the second study by Milan *et al*. (2003), subjects were drawn from a twin cohort and a single self-reported weight was the basis of their assessment of BMI in relation to incident BCC over the next 23 years. That is, no account of weight change or assessment of latent effect of BMI on disease was possible. Our study reported here was a prospective, population-based study of anthropometric measures and BCC and thus susceptible to neither recall nor selection biases. It is the first study to have used objectively measured height and weight, together with measured waist and hip circumference in relation to BCC, and the only study to have examined weight change. Also the potential influences of most known risk factors for BCC were thoroughly assessed.

Limitations of the study include reliance on self-report of BCC prior to 1986, and availability of only a single height measurement in 1992. In older adults (age 60+), height can decrease with age due to spinal deformity and thinning of the intervertebral discs [35]. This decrease in height has been reported to range from 0.5–1.5 cm/decade [35], to 2.5–5 cm/decade [36]. We could therefore expect that there would have been some change in height for the 26.9% of study participants aged over 60 years (in 1992), however we would expect this change in height to be modest over the 4.5 year follow-up period.

## Conclusion

Adherence to WHO guidelines for BMI, waist circumference, waist/hip ratio appears not to be associated with occurrence of BCC, though there may be a positive association between weight gain and occurrence of BCC in women that warrants further investigation.

## Competing interests

The author(s) declare that they have no competing interests.

## Authors' contributions

CO performed the statistical analysis, and drafted the manuscript. AG participated in the design and coordination of the study. CO, AG and MH were involved in critically revising the manuscript. NP advised on statistical analysis and interpretation of the data. All authors read and approved the final manuscript.

## Pre-publication history

The pre-publication history for this paper can be accessed here:



## Figures and Tables

**Table 1:** Height, weight, BMI, waist circumference, waist/hip ratio and risk of BCC at baseline (1992)

	Males					Females				
	
	BCC		No BCC		PPR (95% CI) ^a^	BCC		No BCC		PPR (95% CI) ^a^
	N	%	N	%		N	%	N	%	
Weight quartile										
Q1	22	25	109	25	1.0	20	25	152	25	1.0
Q2	22	25	112	25	1.2 (0.7–2.0)	21	26	157	25	0.9 (0.5–1.6)
Q3	26	30	108	24	1.4 (0.8–2.2)	13	16	162	26	0.6 (0.3–1.1)
Q4	17	20	116	26	0.9 (0.5–1.0)	26	33	150	24	1.1 (0.7–1.9)
n = 1223					*p *= 0.45					*p *= 0.14
										
Height quartile										
Q1	27	34	90	22	1.0	21	29	136	24	1.0
Q2	13	17	100	25	0.6 (0.3–1.1)	19	26	140	25	1.0 (0.6–1.8)
Q3	18	23	111	28	0.8 (0.5–1.3)	12	16	144	26	0.8 (0.4–1.5)
Q4	21	27	100	25	1.3 (0.8–2.1)	21	29	139	25	1.4 (0.8–2.5)
n = 1112					*p *= 0.07					*p *= 0.31
										
BMI category (kg/m^2^)										
<25	30	38	142	36	1.0	33	45	286	51	1.0
25–29.9	38	48	192	48	1.0 (0.6–1.4)	23	32	179	32	0.9 (0.6–1.5)
≥ 30	11	14	66	17	0.8 (0.4–1.5)	17	23	92	17	1.2 (0.7–2.1)
n = 1109					*p *= 0.76					*p *= 0.64
										
Waist category (cm)										
<94 (M); <80 (F)	31	36	197	45	1.0	31	39	271	44	1.0
94–101.9 (M); 80–87.9 (F)	30	35	114	26	1.3 (0.9–2.0)	49	61	343	43	0.9 (0.6–1.5)
102 + (M); 88 + (F)	26	30	129	29	1.0 (0.6–1.5)	0		0		
n = 1221					*p *= 0.31					*p *= 0.79
										
Waist/hip ratio category										
≥ 1.0 (M); ≥ 0.85 (F)	78	90	397	90	1.0	64	80	526	88	1.0
> 1.0 (M); > 0.85 (F)	9	10	43	10	0.8 (0.4–1.4)	16	20	87	12	1.0 (0.6–1.7)
n = 1220					*p *= 0.41					*p *= 0.90

^a ^Adjusted for age; waist/hip ratio category adjusted for age and skin colour

**Table 2:** Height, weight, BMI, waist circumference, waist/hip ratio and weight change and incidence of BCC 1992–1996

	Males					Females				
	
	BCC		No BCC		RR (95% CI) ^a^	BCC		No BCC		RR (95% CI) ^a^
	N	%	N	%		N	%	N	%	
Weight quartile										
Q1	23	30	97	24	1.0	17	21	141	25	1.0
Q2	15	20	107	26	0.7 (0.5–1.1)	19	24	146	26	1.1 (0.6–1.8)
Q3	21	28	100	24	0.9 (0.6–1.3)	17	21	152	27	0.9 (0.5–1.5)
Q4	17	22	106	26	1.0 (0.6–1.5)	27	34	131	23	1.4 (0.9–2.4)
n = 1223					*p *= 0.29					*p *= 0.22
										
Height quartile										
Q1	18	27	84	23	1.0	19	25	125	24	1.0
Q2	15	23	92	25	1.3 (0.8–2.1)	20	27	127	25	1.0 (0.6–1.6)
Q3	21	32	98	26	1.5 (1.0–2.3)	16	21	133	26	1.0 (0.6–1.7)
Q4	12	18	97	26	0.9 (0.6–1.4)	20	27	127	25	1.3 (0.8–2.3)
n = 1112					*p *= 0.16					*p *= 0.62
										
BMI category (kg/m^2^)										
<25	23	35	131	35	1.0	35	47	262	51	1.0
25–29.9	34	52	178	48	0.9 (0.6–1.4)	26	35	166	33	0.7 (0.5–1.1)
≥ 30	9	14	61	17	1.0 (0.6–1.7)	14	19	82	16	1.2 (0.7–2.0)
n = 1109					*p *= 0.94					*p *= 0.15
										
Waist category (cm)										
<94 (M); <80 (F)	27	36	182	45	1.0	26	33	252	45	1.0
94–101.9 (M); 80–87.9 (F)	25	33	106	26	0.9 (0.6–1.3)	53	67	312	55	1.0 (0.8–1.4)
102 + (M); 88 + (F)	24	32	117	29	1.0 (0.6–1.5)	0		0		
n = 1221					*p *= 0.83					*p *= 0.71
										
Waist/hip ratio category										
≥ 1.0 (M); ≥ 0.85 (F)	69	91	365	90	1.0	63	80	485	86	1.0
> 1.0 (M); > 0.85 (F)	7	9	40	10	0.9 (0.5–1.5)	16	20	78	14	1.1 (0.7–1.7)
n = 1220					*p *= 0.53					*p *= 0.85
Weight change (kg)										
<-4	10	14	29	18	1.2 (0.6–2.5)	14	19	44	9	1.5 (0.8–2.7)
-4–3.9	48	66	232	65	1.0	37	51	311	62	1.0
4–10	15	21	85	24	1.1 (0.6–1.9)	18	25	115	23	1.4 (0.8–2.5)
10+	0	0	13	4		4	6	29	6	1.7 (0.5–5.6)
n = 1004					*p *= 0.63					*p *= 0.52

^a ^Adjusted for age, and history of BCC

Weight quartile and waist circumference category adjusted for age, history of BCC and hair colour

Height quartile and BMI category adjusted for age, history of BCC and eye colour

Weight change category adjusted for age, history of BCC, weight at baseline, hair and eye colour
